# Evaluation of Poultry Manure: Combination of Phosphorus Recovery and Activated Carbon Production

**DOI:** 10.1021/acsomega.2c00975

**Published:** 2022-06-09

**Authors:** Nurdan Sevde Topcu, Gozde Duman, Hayati Olgun, Jale Yanik

**Affiliations:** † Chemistry Department, Ege University, 35100, Bornova, İzmir, Turkey; ‡ Solar Energy Institute, Ege University, 35100, Bornova, İzmir, Turkey

## Abstract

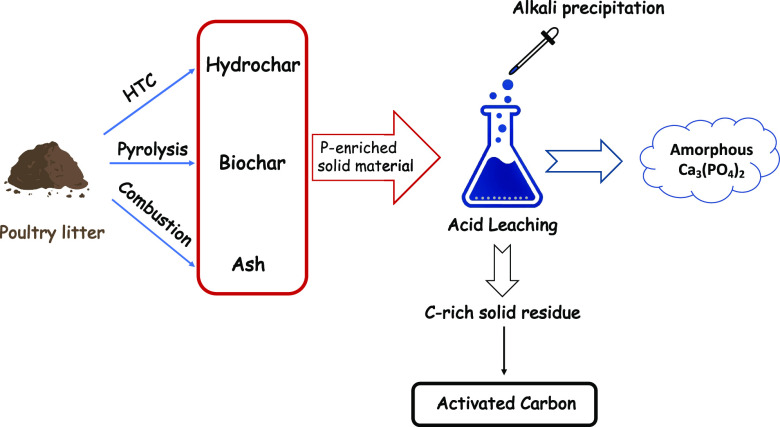

Intensive growth of poultry production leads to generation of a large-scale accumulation of wastes, which is a critical concern for poultry farming. An environmentally friendly and effective solution is still being sought for sustainable management of poultry manure. In this study, evaluation of poultry manure both as a carbon source for production of solid fuels and activated carbon and as a phosphorus source has been investigated. The study focuses on the following: (1) biochar and hydrochar production under different process conditions for production of carbon-rich fuel from poultry manure; (2) phosphorus recovery by acid leaching–alkali precipitation from manure ash, biochar, and hydrochar; and (3) activated carbon production from acid-leached hydrochar and biochar. The results reveal that production of biochar and hydrochar is not a promising method for upgrading laying hen manure into an energy-dense solid fuel. Phosphorus in ash and chars was recovered as amorphous calcium phosphate with yields of 57.3–48.5% by acid leaching–alkali precipitation. Untreated and acid-leached chars were subjected to a chemical activation process with KOH and ZnCl_2_ to produce activated carbon. Due to the catalytic effect of inorganics in chars, the KOH activation resulted in a very low yield of activated carbon. The surface areas of activated carbons prepared using ZnCl_2_ were comparable to activated carbons derived from typical biomass using ZnCl_2_.

## 1 Introduction

Phosphorus (P) is an essential element for human nutrition, but the supply of P raw materials is provided by few countries; some of which are located in politically instable regions. Phosphate rock, which is the single source for P, has been considered as critical raw materials by the European Union (EU) (EU Report COM/2014/0297) due to its low substitutability. In the European Union (EU), special attention is paid to critical raw materials under sustainable development principles. In order to achieve P security in the national economy, actions for promoting proper management of P resources have been started in developed EU countries.^1^ Because of this, phosphorus recovery has recently received significant technical and scientific interest. Currently, several ways to recover phosphorus from different waste streams have been defined in some of EU countries such as Germany and Switzerland.^1^ Phosphorus recovery from wastes rich in phosphorus, such as poultry manure, meat, bone meal, sewage sludge, and their incineration ashes, is one of the most promising ways to improve the P resource security.

Meanwhile, since the vast demand of meat and egg leads to the production of larger amounts of poultry manure worldwide, there is a growing concern about its disposal and/or its safe and efficient use. The conventional method for disposal is direct land application as a fertilizer and soil conditioner, but they are now under pressure and no longer a sustainable solution. The most common practices for management of PL are composting, converting into biogas, and combustion with or without pretreatment. A recent review by Drozdz et al.^2^ provides the general knowledge on promising solutions for poultry manure management. Today, the management procedures applied on an industrial scale are as follows: (1) the incineration of poultry manure in thermal plants to generate heat and electricity and (2) production of biogas through anaerobic digestion. But, there are other promising solutions for management poultry manure that are being developed on a laboratory or pilot scale. One of them is pyrolysis to obtain biochar from poultry manure that can be used in a variety of applications, such as sorbents,^3,4^ soil amendment,^5−7^ composting amendment,^8^ and catalysts.^9^

Poultry manure is as a potential renewable source of phosphorus due to its high phosphorus content. While there are an increasing number of papers reporting on the phosphorus recovery from sewage sludge, the literature is still limited on the phosphorus recovery from poultry manure.^10,11^ In these studies, phosphorus was recovered from the poultry manure ash by an acid dissolution–alkali precipitation method that is similar to the P recovery process from sewage sludge ash or biomass ash.

On the other hand, Sun et al.^12^ proposed integration of biochar production with waste management systems to recover and reuse phosphorus. Thus, P in biochar can be reused from P-enriched biochar by using it directly as a soil amendment or by chemical recovery of P. For instance, a phosphate recovery rate of approx. 80–90% was achieved by subsequent acid treatment of the hydrochars derived from manures (cattle, swine, and poultry),^13^ while up to 94.3% of the total digestate phosphate was recovered by acid leaching of the hydrochars.^14^ All of the previous studies have been done with broiler manure, which is a mixture of bedding materials (e.g., wood shavings, saw dust, and rice husk), excreta, feather, feed spills, etc. Unlike other studies, laying hen manure, which is consisted of excreta and a bit of broken down feathers, spilled feed, or broken egg shells, was selected as a feedstock in this study.

The main objectives of the present study are as follows: (1) biochar and hydrochar production under different process conditions for upgrading poultry manure into an energy-dense solid fuel; (2) phosphorus recovery by acid leaching–precipitation from manure ash, biochar, and hydrochar; and (3) activated carbon production from acid-leached hydrochar and biochar. Although, for over 20 years, many researchers have been focused on modification/activation of biochars,^15,16^ there has not been any report on preparation of activated carbon from the acid-leached char residues. The novelty of the present work is to evaluate laying hen manure in combination with phosphorus recovery and activated carbon production.

## 2 Materials and Methods

### 2.1 Materials

The poultry manure (laying hens’ litter) having 25% moisture and its ash were supplied by Gures Energy (Turkey). Ash samples (FA) were collected from cyclone in a fluidized bed combustion system in a commercial incineration plant (Gures Energy). To prevent the microbial decaying during storage, after grinding to 0.5 mm particle size, the poultry manure (PM) was dried in an oven at 105 °C and stored in closed containers.

H_2_SO_4_ (Tekkim, 98% purity) and NaOH (Tekkim, Extra pure) were used for acid leaching–alkali precipitation.

### 2.2 Pyrolysis and Hydrothermal Carbonization

Dried PM samples were pyrolyzed at three different temperatures (300, 400, and 500 °C) in a laboratory-scale vertical fixed bed reactor. The detailed information of the pyrolysis was given in a previous study.^5^ Briefly, after 50 g of poultry manure was loaded to a reactor, the reactor was heated to the desired temperature with a heating rate of 10 °C min^–1^ and held at that temperature for 1 h. The reactor was then cooled down to room temperature under N_2_ flow. The solid product (biochar) from pyrolysis was weighed and then stored in sealed containers. Hydrothermal carbonization (HTC) was carried out using an autoclave (Buchi Glassuster Limbo model, 500 mL volume) with a magnetic stirrer under autogenic pressure. In a typical run, a mixture of poultry manure and deionized water at the 1:5 solid-to-water ratio was loaded into the reactor. After sealing and purging with N_2_ to remove residual air, the autoclave was heated to the desired temperature with a heating rate of 5 °C min^–1^ and held at this temperature for the required duration (0 and 60 min). At the end of the process, the autoclave was cooled rapidly to room temperature. After venting the gaseous products into the atmosphere, the autoclave content was filtered to separate the solid product (hydrochar) from process water. The obtained hydrochar was dried at 105 °C for 24 h and then stored in sealed containers. The biochars were named as “PC-process temperature”. The hydrochars were named as “HC-process temperature-duration”.

Mass and carbon yields were calculated as:
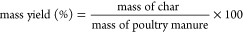

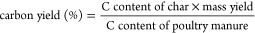


### 2.3 Acid Leaching and Alkaline Precipitation

Acid leaching method was used to recover the phosphate from ash and chars (biochar/hydrochar). Leaching was performed using different concentrations of H_2_SO_4_ (0.1, 0.3, and 0.5 M) at two different liquid-to-solid ratios (L/S) of 50 and 100 mL/g. In a typical run, ash and char samples were mixed with H_2_SO_4_ under vigorous stirring for two different contact times (2 and 4 h). At the end of the desired contact time, the liquid phase (leachate) was separated by filtering with a filter paper under vacuum. The concentration of phosphorus in leachate was measured by a vanadium molybdate method. Extraction efficiency was calculated as
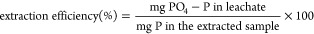


In the precipitation step, 1 M NaOH was added dropwise to the leachate with slow stirring until the pH reached 8, where white/light brown precipitate was formed. The solution was then allowed to age for 4 h for the precipitation reactions to complete. Then, the precipitate was separated by filtration using a filter paper and dried at 100 °C.

### 2.4 Activation of Chars

Both chars and acid-leached chars were subjected to chemical activation. ZnCl_2_ and KOH solution were used in an impregnation ratio of 1:1 (weight of impregnation reagent:weight of char). The impregnated samples with ZnCl_2_ and KOH were pyrolyzed for 1 h at 500 and 700 °C, respectively. After pyrolysis, activated chars were boiled with 10% HCl solution for 1 h, filtered in a vacuum flask, and washed with hot water and finally with cold water to remove the residual potassium and zinc salts from the produced activated chars.

### 2.5 Analysis

The major elements in solid samples were determined by ICP-MS (Agilent 7900) following microwave-assisted acid digestion (0.5 g of sample + 3 mL of H_2_O_2_ + 5 mL of HNO_3_). Elemental analysis of PM and chars was carried out with a LECO CHNS 932. The ash and volatile matter content analysis was done according to NREL/TP-510-42622 and ASTM D3175-89a, respectively. The higher heating value (HHV) of the hydrochars/biochars was calculated according to following formula:^17^ HHV = 0.3491C + 1.1783H + 0.1005S – 0.1034O – 0.0151N – 0.0221A. The soil available phosphorus in solid samples was determined using the Olsen method.^18^

X-ray powder diffraction (XRD) was used to examine the main crystalline phases in the samples. The XRD measurements of samples were performed on a Malvern Panalytical (Aeris Research Edition) using a Cu Kα (40 kV × 15 mA) and a PIXcel3D 2D solid-state hybrid pixel detector. Data were collected in the 2θ range from 5 to 80° in steps of 0.01 using 63 s per step.

The BET (Brunauer–Emmett–Teller) surface area measurements of activated carbons were obtained from nitrogen adsorption isotherms at 77 K using a Micrometrics FlowSorb II-2300 surface area analyzer. Prior to measurement of N_2_ adsorption, activated carbons were degassed for 3 h at 300 °C.

The SEM images of activated carbon were taken by a high-resolution scanning electron microscope (JEOL 6510). The images were collected at 20 kV at a magnetization of 2500. Prior to analysis, activated carbons were coated with gold to assess the image quality.

## 3 Results and Discussion

### 3.1 Poultry Manure as an Energy Source

Conversion of poultry manure into biochar/hydrochar might be a feasible approach for their disposal and for obtaining suitable energy feedstocks. The yield and properties of biochars/hydrochars strongly vary depending on carbonization conditions. Reaction duration and temperature are the most significant parameters for HTC, whereas temperature is the main parameter that affects biochar yields and properties. Therefore, HTC experiments were carried out to determine the effects of reaction time and temperature on the mass yield and fuel characteristics of hydrochars derived from PM. HTC parameters were selected according to our previous study.^5^ In the pyrolysis experiments, the effect of reaction temperature on the mass yield and characteristics of biochars derived from PM was investigated.

**Figure 1:**
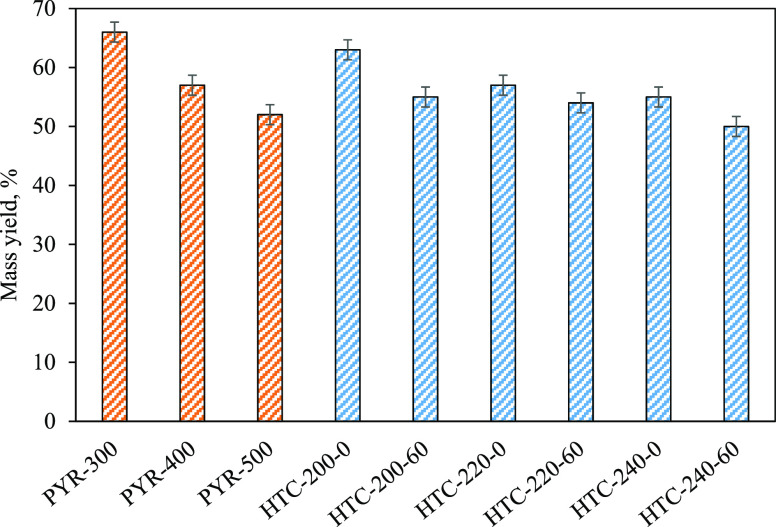
Mass yields from HTC and pyrolysis of poultry manure under different process conditions.

In the case of HTC, the effect of reaction time on hydrochar yield varied with temperature (Figure 1). Although, at 220 °C, the mass yield of hydrochar did not change significantly by increasing the duration, it decreased by increasing the duration at the temperatures of 200 and 240 °C.

The decrease in mass yield as the temperature and duration are both increased might be due to the enhanced decomposition of PL or through decomposition of secondary char. The fact that there was no influence of duration at 220 °C might be due to the formation of secondary char^19^ besides degradation of biomass for the duration of 60 min. The higher C content of HTC-220-60 than HTC-220-0 (Table 1) also supports this idea. Based on the information in previous studies, it can be noticed that there is no common consensus about the effect of duration on the HTC process. The hydrochar yields obtained in this study are comparable to the previous studies carried out with livestock wastes, such as dairy manure,^20^ poultry manure, swine manure, dairy cattle manure,^13^ cow manure,^21^ and broiler poultry litter.^5^

**Table 1:** Some Properties of Poultry Manure and Its Biochars/Hydrochars

	ultimate analysis (wt %)					atomic ratio					
sample	C	H	N	S	O^a^	O/C	H/C	ash (wt %)	HHV (MJ/kg)	ED^b^	C yield (%)
PM	34.4	4.6	2.9	0.1	21.3	0.46	1.60	36.7	14.4		
PC-300	35.4	2.8	3.3		19.0	0.40	0.95	39.5	12.8	0.89	67.9
PC-400	34.0	2.0	2.6		17.6	0.39	0.71	43.8	11.4	0.79	56.3
PC-500	31.0	0.7	1.9		16.7	0.40	0.27	49.7	8.8	0.61	46.9
HC-200-0	31.0	3.8	2.2		29.5	0.71	1.47	33.5	11.5	0.80	56.8
HC-200-60	31.0	3.8	2.0		30.5	0.74	1.47	32.7	11.4	0.79	49.6
HC-220-0	32.2	3.6	1.9		27.6	0.64	1.34	34.7	11.9	0.82	53.4
HC-220-60	34.7	3.6	2.1		22.7	0.49	1.24	36.9	13.2	0.92	54.5
HC-240-0	34.2	3.6	1.9		24.6	0.54	1.26	35.7	12.9	0.89	54.7
HC-240-60	34.4	3.3	2.2		20.0	0.44	1.15	40.2	12.9	0.90	50.0

a O (%) = 100 – (C + H + N + S + ash).

b ED (energy density) = HHV_char_/HHV_PM_.

In the case of pyrolysis, the biochar yield gradually decreased from 66 to 52% with the increase in temperature from 300 to 500 °C (Figure 1). Although the temperature of pyrolysis was much higher than that of HTC, the yield of biochars did not significantly differ from hydrochar yields. This is also in line with the findings of Liu et al.^22^ They reported that the yields of biochar obtained from pig manure varied from 57.8 to 42.9% over a temperature range of 300–500 °C and the hydrochar yield varied from 56.7 to 43.7% at a temperature range of 180–300 °C. In contrast, for the lignocellulosic biomasses, such as Eucommia,^23^ olive tree and vineyard prunings,^24^ grape pomace,^25^ and miscanthus,^26^ it was reported that biochar yields were higher than hydrochar yields. Also, it was postulated that the extent of degradation of biomass polymers (i.e., hemicellulose, cellulose, and lignin) significantly depended on the reaction medium and biomass constituents were less stable under hydrothermal conditions. Due to the decomposition of biomass in HTC being initiated by hydrolysis, activation energy is lower than most of the pyrolytic decomposition reactions. But, in this study, the used litter (from laying hen) had no lignocellulosic bed material. It was also noted that char (biochar/hydrochar) yields were found to be higher than most reported char yields from various types of biomass due to the high ash content. It is well-known that the HTC process reduces the ash content of char due to the dissolution of inorganics in biomass. But, in this study, 70–80% of ash in PM was retained in the hydrochars (reason will be discussed in the following sections).

The variance in the main fuel characteristics of biochars/hydrochars produced under different process conditions is given in Table 1. It is unexpected that the fraction of carbon in biochar was decreased by increasing the pyrolysis temperatures due to the high ash content. But, if we consider C contents on an ash free basis (daf), it was seen that the C content in biochar slightly increased with increasing pyrolysis temperature, passing from 58.5% (daf) at 300 °C to 61.6% (daf) at 500 °C, which were higher than the original feedstock (46.5%, daf). During HTC, the carbon content in hydrochars also increased (from 46.6 to 57.5%, daf) with an increase in the process severity. Both biochars and hydrochars (except HTC-200) exhibited low H/C–O/C ratios compared to the PM.

Knowing the elemental composition of the PM and the elemental composition and mass yield of the char (on ash free basis), the fate of carbon and nitrogen was calculated by mass balances. The C recovery in biochar decreased from 67.9 to 46.9% as the temperature increased, which might be attributed to the enhanced decomposition of PM and/or secondary decomposition of char. On the other hand, the C recovery in hydrochars (49.6–56.8 wt %) did not significantly change under the process conditions studied.

In the case of distribution of nitrogen in PM, between 75 and 34 wt % of nitrogen was retained in biochar, the loss of N from PM was greater at higher pyrolysis temperatures, which might be an increase in NH_3_ evolution by fragmentation of organic N. On the other hand, the amount of N in PM (36–48 wt %) retained in hydrochar was found lower in comparison to biochars produced at low temperatures. This difference might also be due to the hydrolysis of protein and nitrogenous compounds under hydrothermal conditions.

Although pyrolysis and HTC are widely applied in energy densification of biomass, in our case, the energy densification ratio was found to be smaller than 1, resulting from lower HHV values of chars than poultry manure. We concluded that productions of biochar and hydrochar are not promising methods for upgrading laying hen manure into an energy-dense solid fuel. In contrast, Mau and Gross,^27^ who compared the production of biochar and hydrochar from broiler manure in terms of char quality, energetics, and gas emissions, suggested that the production of biochar and hydrochar is the most suitable way for the use of poultry manure as an energy source.^27−29^ The difference in manure constituents could explain this contrary result. They used the manure containing mainly broiler excretions mixed with some bedding materials and feathers, while the manure used in this study was only consisted of laying hens’ excretions having a high Ca content.

### 3.2 Poultry Manure as a Source of Phosphorus

#### 3.2.1 Phosphorus Contents of Poultry Manure, Its Ash, and Chars

The main components of samples were phosphorous, calcium, potassium, magnesium, and sodium (Table 2). Incineration and pyrolysis led to an increase in most of the elements; the P concentration was increased by 1.2–1.9 times by pyrolysis, depending on the weight loss of manure. About 90–99% of the P present in manure remained in the hydrochars (Table 3).

**Table 2:** Main Inorganics in Poultry Manure, Its Ash, and Chars (g/kg)

sample	Na	Mg	Al	Si	P	K	Ca	Mn	Fe	Zn
manure	4.7	9.1	0.6	0.7	21.7	23.4	106.8	0.4	1.0	0.5
FA	23.5	41.0	2.9		92.2	53.7	294.3	1.9	4.6	1.6
PC-300	6.2	11.6	0.7	0.5	26.8	33.0	152.5	0.5	2.3	0.8
PC-400	8.7	15.8	1.0	0.8	37.6	43.6	189.8	0.7	2.9	1.4
PC-500	9.1	17.8	1.0	0.7	40.2	46.2	199.3	0.8	2.0	1.0
HC-200-0	1.5	6.9	0.6	0.9	26.3	7.0	147.6	0.5	1.0	0.6
HC-220-0	0.7	5.3	0.6	1.0	29.4	2.0	158	0.5	1.0	0.6
HC-220-60	0.6	7.6	0.8	0.7	35.7	0.9	195.7	0.6	1.3	0.8
HC-240-0	0.6	6.0	0.6	1.3	31.5	1.2	161.7	0.5	1.2	0.6
HC-240-60	0.5	7.1	0.8	0.8	31.7	0.6	161.9	0.5	1.3	0.7

**Table 3:** Recovery of P in Hydrochars (%)

HC-200-0	HC-220-0	HC-220-60	HC-240-0	HC-240-60
93	94	99	97	89

The phosphorus content of the resulting hydrochar varied slightly depending on HTC conditions. The phosphorus content tended to slightly increase with increasing reaction severity; in the case of the highest temperature and time, phosphorus in the resulting hydrochar decreased. In contrast to previous studies related to HTC of spent coffee grounds,^29^ poultry litter,^28^ sewage sludge,^30^ and microalgae,^31^ majority of phosphorus in manure was accumulated within the solid hydrochars in this study. This could be attributed to the combination of P-containing species with Ca to form insoluble phosphates. Heilmann et al.^13^ postulated that the presence of high concentrations of multivalent metal cations such as aluminum, calcium, magnesium, and iron may form insoluble phosphates that are entrapped on or within growing hydrochars. Yu et al.^32^ also reported that P minerals were highly associated with the predominance of Al, Ca, and Fe in hydrochar-derived sewage sludge. It was reported that the precipitation of phosphorus salts in hydrochar, such as calcium phosphate, magnesium phosphate, and magnesium ammonium phosphate (struvite), led to immobilize P in the hydrochar.^33^ The precipitation of phosphorous salts in hydrochar is dependent upon the inorganic content of the feedstock and pH of HTC solution. As a general principle, it is suggested that a large amount of polyvalent cations leads to a more effective P immobilization on hydrochar due to the low solubility of their phosphates.^14^ In our study, due to the presence of high calcium ions in poultry manure, Ca–P interactions may occur to form insoluble calcium phosphate salts.^12^ But, for a longer reaction time, at a high temperature (240 °C), the formation of insoluble multivalent metal phosphates increased.

Although the thermal treatment concentrated phosphorus in the char and ash, not all the phosphorus was bioavailable, similar to previous studies.^30,34^ The extracted P species by the Olsen method are orthophosphates. The efficiency of soil available P extraction decreased with increasing pyrolysis temperature (Table 4). It is possible that physical constraints as a result of carbon condensation during pyrolysis prevented orthophosphates from being extracted^30^ or converted the orthophosphates into water-insoluble P species. Compared to biochars, hydrochars contained less amount of bioavailable P as water-soluble phosphorus species were already transferred into the process water during HTC. In contrast, Yang et al.^35^ stated that organic acids such as humic acid, which is produced during HTC of biomass, enhanced P bioavailability. This contradictory result can be attributed to the high alkalinity of the resulting hydrochar obtained from poultry manure.

**Table 4:** Content of Soil Available P of the Poultry Manure Ash and Chars (mg/g)

sample	available P		sample	available P
FA	0.02		HC-200-0	6.21
PC-300	7.76		HC-200-60	4.47
PC-400	7.02		HC-220-0	5.18
PC-500	5.25		HC-220-60	2.61
			HC-240-0	4.6
			HC-240-60	0.86

**Figure 2:**
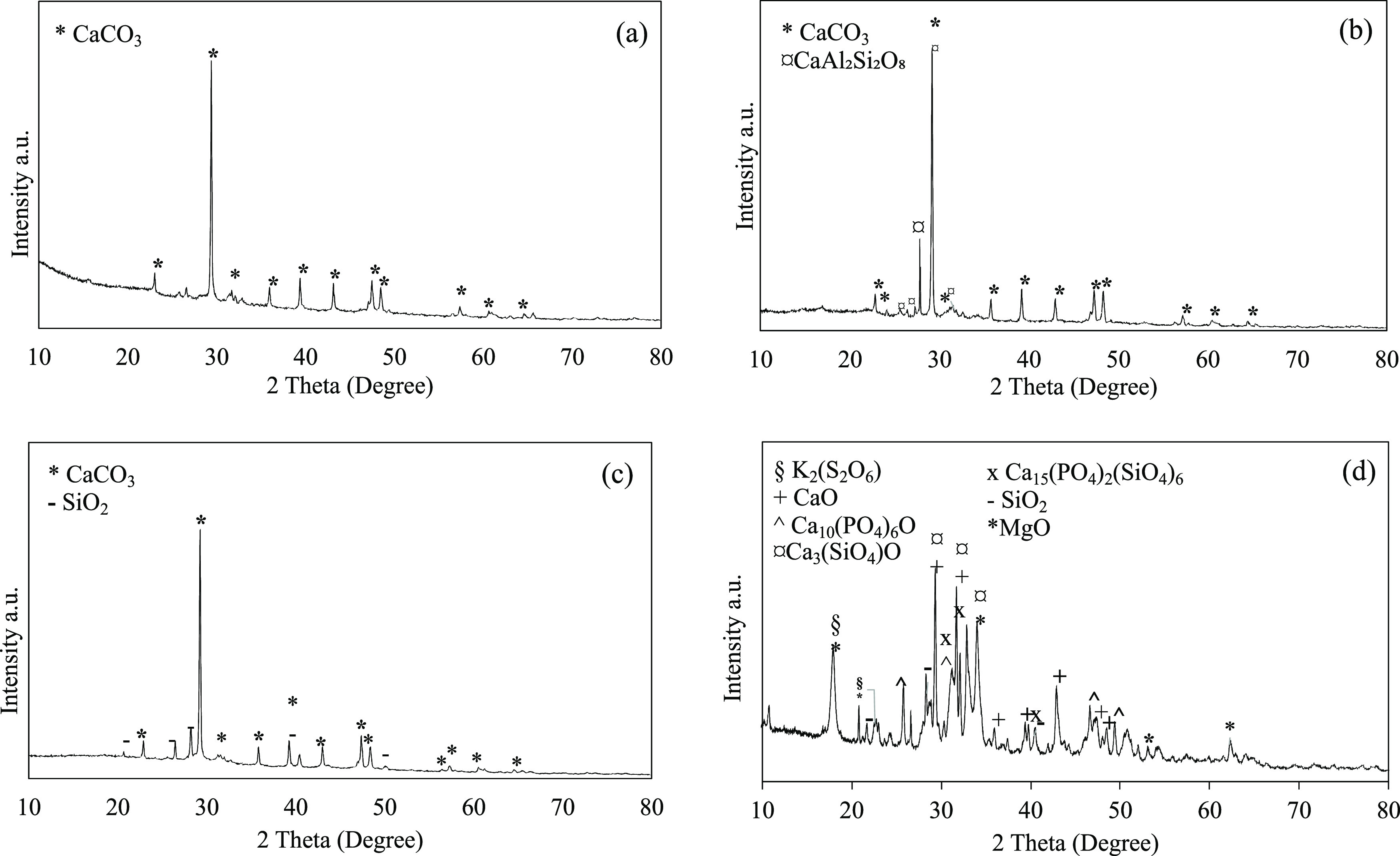
XRD spectra of (a) poultry litter, (b) HC-220-60, (c) PY-300, and (d) fly ash (FA).

To gain insights into the crystalline species of P in the manure, ash, and chars, XRD was applied. As seen in Figure 2b, CaO, which is the main component in the ash, can be found in the form of four primary phases as free calcium oxide (CaO) or in the form of silicate (Ca_3_(SiO_4_)O), phosphate (Ca_10_(PO_4_)_6_O), and complex phase of phosphate and silicate (Ca_15_(PO_4_)_2_(SiO_4_)_6_) while the main crystalline phase is CaCO_3_ in manure. On the other hand, a new SiO_2_ crystalline phase was formed during pyrolysis while CaAl_2_Si_2_O_8_ was observed after hydrothermal carbonization.

#### 3.2.2 Phosphorus Recovery by Extraction–Precipitation

As known, manure is one of the potential P resources. P was concentrated in ash and chars following thermal treatment; however, there is one barrier that prevents their direct use as a P fertilizer, namely, the bioavailability of phosphorus in ash/chars is not favorable, constituting a little part of the total phosphorus. They can be used as a concentrated phosphorus source for further extraction and transformation. Phosphorus recovery generally involves two steps: extracting P from solids to liquid and producing P fertilizer by precipitation. Acidic extraction is the widely used method for P recovery due to its high extraction efficiency. Many process parameters such as acid concentration, the ratio of acid to solid (L/S), and leaching time have a significant impact on the extraction of P. Although both inorganic and organic acids had high P extraction capacities, it was suggested that sulfuric acid was the most efficient acid from the economic point of view. A series of experiments were performed to determine the optimal conditions in the extraction of P from ash and chars (Supporting Information). Except for leaching of FA at the 50 mL/g L:S ratio, the extraction efficiency ranged between 88 and 99%. In the case of FA, an L/S ratio of 50 mL/g was not effective for P extraction. This is because the phosphorus recovery is dependent on the end pH of extraction solutions.^36^ The pH of solute after 4 h reached 7.2 in leaching of FA at the 50 mL/g L:S ratio. The pH of the final solute showed that the amount of acid was not sufficient for extraction of phosphate and the acid in the environment was only consumed in the neutralization of alkaline oxides. For FA leaching, 0.1 M H_2_SO_4_, 100 mL/g L:S ratio, and 4 h were chosen as the optimum conditions. In the case of biochar/hydrochar, the extraction conditions had no significant effect on phosphorus recovery. The reason might be due to the formation of gypsum on the solid surface, which can restrict the further contact between acid and solid. The XRD patterns of acid-leached biochar/hydrochar also supported the presence of gypsum (Supporting Information).

Considering acid consumption, 0.1 M H_2_SO_4_, 50 mL/g L:S ratio, and 4 h were selected as the optimum conditions for biochar/hydrochars. Compared to biochars, higher extraction efficiencies were obtained from hydrochars. The reason was related to P speciation in chars depending on the carbonization type (pyrolysis or HTC). Huang and Tang,^30^ who studied the effect of pyrolysis and hydrothermal carbonization on the conversion of P species in activated sludge, reported that P species in the biochar was dominated by short-chain polyphosphates, whereas only inorganic orthophosphate existed in the hydrochar. Since the systematic characterization of P species in chars is beyond the scope of this study, P species has not been studied in detail.

The precipitate yields and P recovery based on the initial feedstock (before acid leaching) are given in Table 5. Extraction efficiency in acid leaching is also given in the same table. Although acidic extraction efficiencies are higher than 90%, the final P recovery from ash/chars is about half of the phosphorus in the initial feedstock. The low recovery of phosphorus as a solid precipitate might be due to the formation of soluble phosphorus salts during alkaline precipitation.^37^ As known, some hydrogen phosphates, such as Ca(H_2_PO_4_)_2_, are soluble as well as phosphate salts of sodium, potassium, and ammonium.

**Table 5:** Extraction Efficiency, Precipitation Yield, and P Recovery

feedstock	extraction efficiency (%)	precipitation yield^a^ (wt %)	P in precipitate (mg/g)	P recovery^a^ (%)
FA	94.5	27.5	192.2	57.3
PC-300	91.0	11.4	113.9	48.5
HC-220-60	98.0	10.4	179.2	52.2

a Based on feedstock.

**Figure 3:**
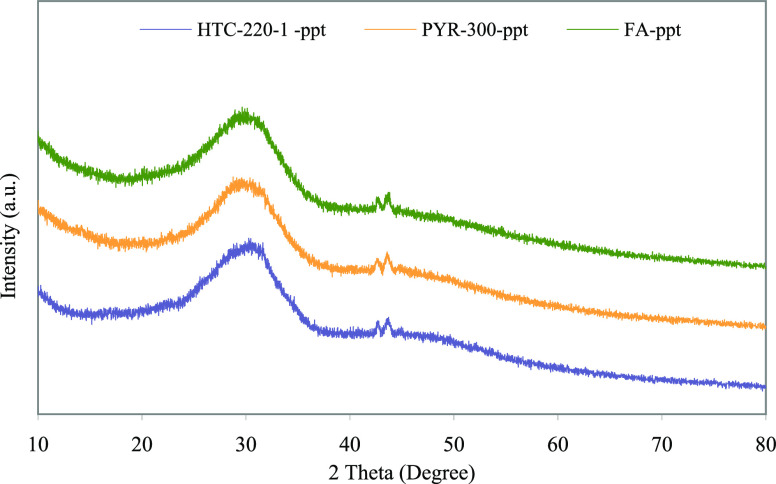
XRD patterns of precipitates.

The broad and diffusive patterns of XRD at about 30° (Figure 3) showed that the treatment by H_2_SO_4_ extraction–alkali precipitation yielded a precipitate of amorphous calcium phosphate (Ca_3_(PO_4_)_2_).

### 3.3 Poultry Manure as an Activated Carbon Precursor

Due to their high acid content, biochar/hydrochar cannot be used as fuels after phosphorus recovery. The better option might be their use as precursors in activated carbon production. Recently, much attention has been focused on the use of biochar-obtained wastes as carbon precursors in activated carbon production due to its homogeneous character compared to raw wastes. Ahmed et al.^38^ also reported that up to 70% reduction in chemical usage was achieved by using of biochar instead of biomass. In the literature, there are numerous studies on chemical activation of biomass chars (Supporting Information). Unlike studies in the literature, acid-leached biochar/hydrochar was used to produce activated carbon in this study. After acid leaching, the char residues were directly subjected to chemical activation without washing with water. For comparison purposes, the unleached chars obtained under optimized conditions were also subjected to chemical activation. Sample names ePYR-300 and eHC-220-60 refer to the acid-leached biochar and hydrochar, respectively. In the activation of chars with KOH or ZnCl_2_, the following processes take place: in the impregnation step, the chemical agent diffuses into the internal of the chars, and then during post-pyrolysis, elimination of the tar deposits and char activation occur.

KOH is commonly used as an activation agent to obtain activated carbon from chars having high surface areas.^37^ In contrast to previous studies,^38,39^ KOH activation of both raw and acid-leached chars resulted in very low mass yields, ranging from 1.2 to 5.7 wt % of both untreated and acid-leached chars. This might be due to the intensive gasification caused by the catalytic effect of inorganic in chars because, as is known, KOH activation is naturally associated with a gasification reaction (KOH + C → K + K_2_CO_3_ + H_2_).^40^ On the contrary, in activation with ZnCl_2_, a high yield of activated carbon was obtained due to some aromatic condensation reactions. The textural properties of activated carbons obtained by chemical activation with ZnCl_2_ are given in Table 6. The product yield and ash content of activated carbons are also presented in the same table. The ash contents of activated carbons were significantly lower than those of carbon precursors because of acid and water washing. The surface areas of the prepared activated carbons from the acid-leached char residues derived from poultry manure are comparable to those of activated carbons derived from biomass using ZnCl_2_, such as from hazelnut husk (1369 m^2^/g),^41^ from tobacco stem (977.2 m^2^/g),^42^ and from silver berry seeds (1109 m^2^/g).^37^ In the case of biochars, activated carbon produced from acid-leached biochar had a lower micropore area than that from raw biochar. Removal of inorganics during acid leaching may lead to inhibition of the formation of micropores. Alkali metals, notably K, in raw biochar can also act as a self-activator for pore development.^43^ On the other hand, activated carbons obtained from hydrochars had a higher surface area than those from biochars. This is probably due to the higher oxygenated functional groups on the surface of hydrochars, which provides efficient chemical activation,^44^ although the K content was low.

**Table 6:** Yield and Some Properties of Activated Carbons Obtained by Chemical Activation with ZnCl_2_

carbon precursor	yield^a^ (wt %)	ash (wt %)	BET surface area (m^2^g^–1^)	micropore area (m^2^g^–1^)	micropore volume (cm^3^g^–1^)	% of *S*_micro_ in *S*_BET_
PYR-300	39.2	23.5	1059	791	0.350	74.6
ePYR-300	45.7	26.5	605	393	0.182	64.9
HC-220-60	39.1	5.7	1142	421	0.203	36.9
eHC-220-60	51.6	16.2	1224	391	0.188	31.9

a Calculated based on char weight.

Although ZnCl_2_ showed a good activation performance in the preparation of activated carbon from acid-leached chars derived from poultry manure, the environmental contamination concerns of using ZnCl_2_ still need to be noticed and further studies are needed to recover and reuse the activating agent.

**Figure 4:**
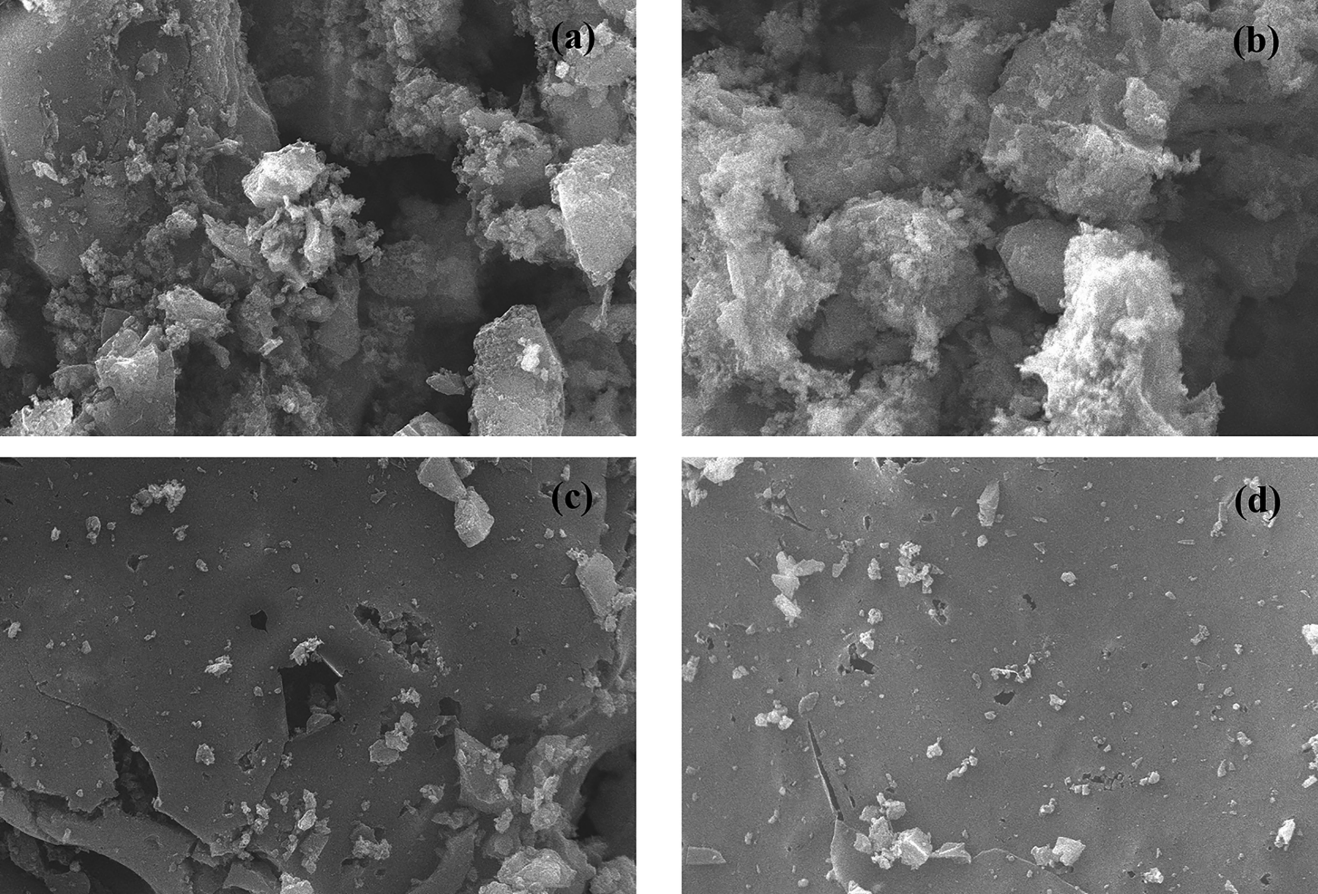
SEM images of activated carbons obtained from (a) PYR-300, (b) ePYR-300, (c) HC-220-60, and (d) eHC-220-60 (at a magnification of 2500).

The SEM images of the surface morphologies of activated carbon are demonstrated in Figure 4. There was an apparent difference in the morphology of activated carbons obtained from biochars and hydrochars. Activated carbon derived from biochars consisted of small particles with irregular shape, whereas activated carbons derived from hydrochars had a smooth surface. It can be concluded that the morphology of activated carbon resembles that of their parent precursor. During pyrolysis, the carbon structure is partially damaged and a vesicle-like structure is formed as a result of the release of volatile matter.^45^ On the other hand, hydrochar with a rough structure is usually formed due to repolymerization of organic products during HTC.^46^

## 4 Conclusions

In this study, conversion of poultry manure into value-added products, namely, phosphorus salt and activated carbon, was achieved by combining thermal and chemical treatments. The results showed that the productions of biochar and hydrochar are not promising methods for upgrading poultry manure into an energy-dense solid fuel. However, the P concentration was increased by 1.2–1.9 times by pyrolysis, while HTC retained most of the phosphorus (90%–99%) in hydrochar from the manure. Although thermal treatment concentrated phosphorus in the char and ash, less amount of the phosphorus was bioavailable. By the acid leaching process, over 90% of phosphorus in char/ash was recovered in the acid solution. However, by subsequent alkali precipitation, the final P recovery from ash/chars was low due to the formation of soluble phosphorus salts during alkaline precipitation. The 57.3–48.5% of total P present in the chars and ash was recovered as amorphous calcium phosphate (Ca_3_(PO_4_)_2_). Additionally, the activated carbons could be produced from acid-leached biochar and hydrochar, which have surface areas comparable to activated carbons derived from biomass.

## Supporting Information Available

P extraction under different conditions (Table S1), surface areas of activated carbon obtained from different biomasses and activating agents from previous studies (Table S2), XRD spectrum of the residue after the extraction process (Figure S1), and N_2_ adsorption isotherm of activated carbons (Figure S2) (PDF)
