# Supplementary material for: Evaluation of Poultry Manure: Combination of Phosphorus Recovery and Activated Carbon Production
Source: ACS Omega. 2022 Jun 9;7(24):20710–8. doi: 10.1021/acsomega.2c00975 (PMC9219055; doi:10.1021/acsomega.2c00975)
Supplement: Supplementary file 1 — ao2c00975_si_001.pdf [file ao2c00975_si_001.pdf]

SUPPLEMENTARY MATERIAL

EVALUATION OF POULTRY MANURE: COMBINATION OF PHOSPHORUS RECOVERY AND ACTIVATED CARBON PRODUCTION

Nurdan Sevde Topcu[1], Gozde Duman[1], Hayati Olgun[2], Jale Yanik[1*]

[1] *Ege University, Chemistry Department, 35100, Bornova/İzmir*

[2]*Ege University, Solar Energy Institute, 35100, Bornova/İzmir*

Table S1. Effects of extraction conditions on P extraction

Sample	Acid	L/S, mL/g	Leaching time, h	PO_4-P in solute (g/kg sample)	pH
Fly Ash	0.1 M H_2SO_4	100:1	4	88.1	1.9
	0.3 M H_2SO_4	100:1	4	88.4	0.8
	0.5 M H_2SO_4	100:1	4	87.0	0.5
	0.1 M H_2SO_4	50:1	4	0.59	7.2
	0.1 M H_2SO_4	150:1	4	88.9	1.6
	0.1 M H_2SO_4	100:1	2	81.3	2.5
HC-220-60	0.1 M H_2SO_4	100:1	4	35.6	1.6
	0.3 M H_2SO_4	100:1	4	36.0	0.7
	0.5 M H_2SO_4	100:1	4	37.5	0.5
	0.1 M H_2SO_4	50:1	4	35.0	1.7
	0.1 M H_2SO_4	150:1	4	35.3	1.3
	0.1 M H_2SO_4	100:1	2	35.3	1.3
	0.1 M H_2SO_4	50:1	2	36.5	1.6
PC-300	0.1 M H_2SO_4	100:1	4	22.9	1.3
	0.3 M H_2SO_4	100:1	4	23.2	0.6
	0.5 M H_2SO_4	100:1	4	24.1	0.4
	0.1 M H_2SO_4	50:1	4	22.5	1.8
	0.1 M H_2SO_4	150:1	4	26.0	1.3
	0.1 M H_2SO_4	100:1	2	24.4	1.3
	0.1 M H_2SO_4	50:1	2	24.4	1.8

Table S2. Surface areas of activated carbon obtained from different biomasses and activating agents

Feedstock	Activator	S_{BET} (m^2 g^{-1})
EPFB hydrochar[1]	KOH	1322<br>2239
Sludge biochar[2]	KOH	907.95
Algal hydrochar[3]	KOH	1302
MSW biochar[4]	KOH	49.1
Wood biochar[5]	NaOH	873
Digestate hydrochar[6]	KOH	1945
Corn stalks biochar[7]	H_3PO_4	600
Biochar from tea waste[8]	$KHCO_3$	1981
Fruit peel biochar[9]	KOH	367.10
Ipomoea biochar[10]	KOH	650
Wood biochar[11]	$ZnCl_2$<br>KOH	1093<br>1185
Digestate biochar[12]	$ZnCl_2$	516.67
Wood hydrochar[13]<br>Peanut hull hydrochar[13]	H_3PO_4<br>KOH<br>H_3PO_4<br>KOH	1436<br>222<br>1091<br>571
Coconut shell hydrochar[14]	NaOH	876.14
Rice straw biochar[15]	KOH	772.3
Sludge biochar[15]	KOH	782.6

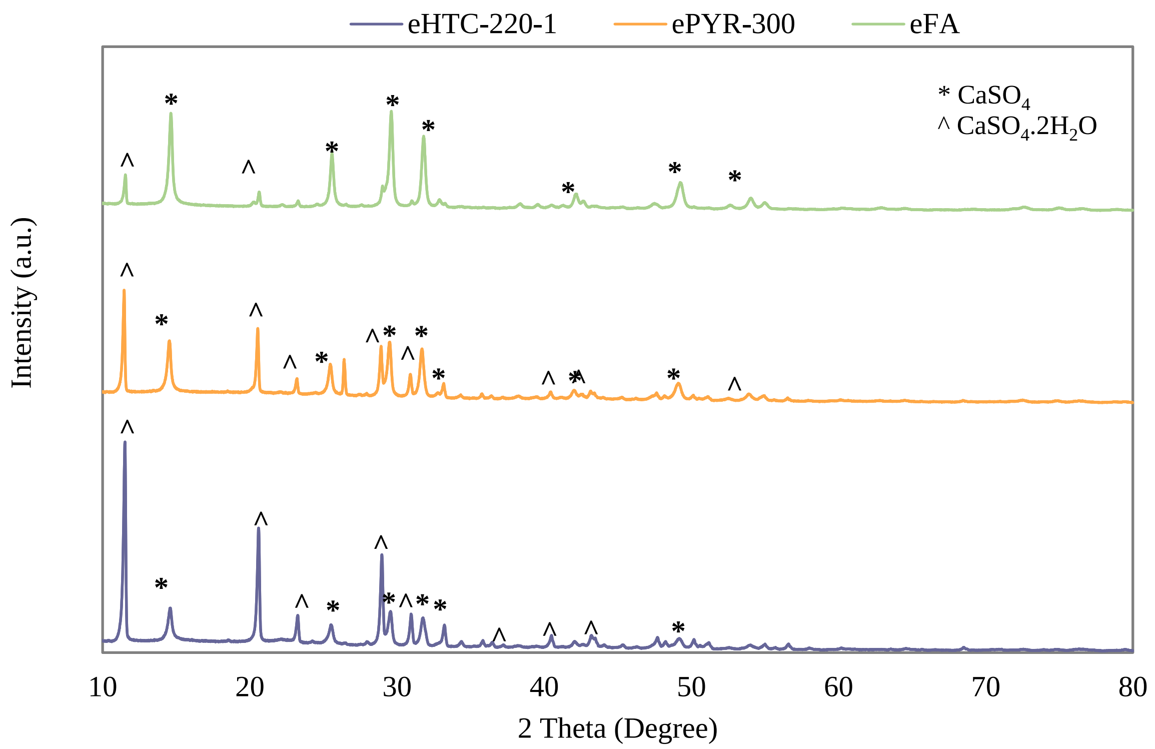

Figure S1. XRD patterns of acid leached biochar and hydrochar

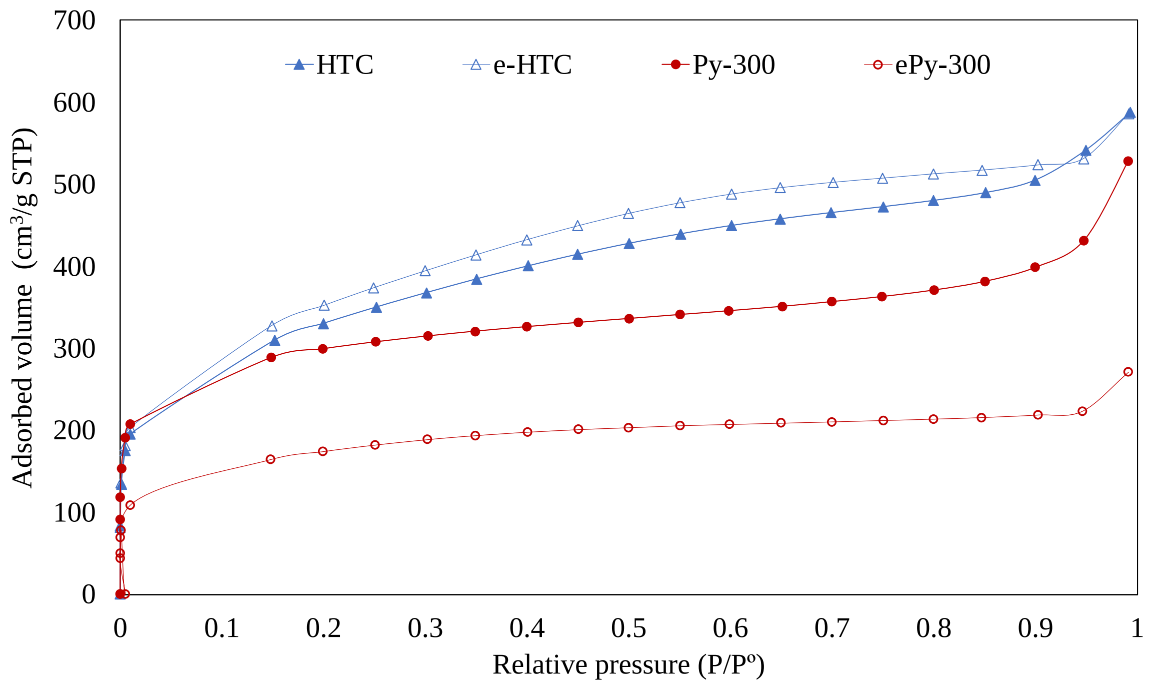

Figure S2. N_2 adsorption isotherm of activated carbons

References

(1) Parshetti, G. K.; Chowdhury, S.; Balasubramanian, R. Biomass Derived Low-Cost Microporous Adsorbents for Efficient CO_2 Capture. *Fuel* **2015**, *148*, 246–254.

(2) Zhang, J.; Shao, J.; Jin, Q.; Li, Z.; Zhang, X.; Chen, Y.; Zhang, S.; Chen, H. Sludge-Based Biochar Activation to Enhance Pb(II) Adsorption. *Fuel* **2019**, *252* (March), 101–108.

(3) Masoumi, S.; Dalai, A. K. Optimized Production and Characterization of Highly Porous Activated Carbon from Algal-Derived Hydrochar. *J. Clean. Prod.* **2020**, *263*, 121427.

(4) Jin, H.; Capareda, S.; Chang, Z.; Gao, J.; Xu, Y.; Zhang, J. Biochar Pyrolytically Produced from Municipal Solid Wastes for Aqueous As(V) Removal: Adsorption Property and Its Improvement with KOH Activation. *Bioresour. Technol.* **2014**, *169*, 622–629.

(5) Ding, Z.; Hu, X.; Wan, Y.; Wang, S.; Gao, B. Removal of Lead, Copper, Cadmium, Zinc, and Nickel from Aqueous Solutions by Alkali-Modified Biochar: Batch and Column Tests. *J. Ind. Eng. Chem.* **2016**, *33*, 239–245.

(6) Zhao, X.; Becker, G. C.; Faweya, N.; Rodriguez Correa, C.; Yang, S.; Xie, X.; Kruse, A. Fertilizer and Activated Carbon Production by Hydrothermal Carbonization of Digestate. *Biomass Convers. Biorefinery* **2018**, *8* (2), 423–436.

(7) Wang, Z.; Wu, J.; He, T.; Wu, J. Corn Stalks Char from Fast Pyrolysis as Precursor Material for Preparation of Activated Carbon in Fluidized Bed Reactor. *Bioresour. Technol.* **2014**, *167*, 551–554.

(8) Li, B.; Huang, Y.; Wang, Z.; Li, J.; Liu, Z.; Fan, S. Enhanced Adsorption Capacity of Tetracycline on Tea Waste Biochar with $KHCO_3$ Activation from Aqueous Solution. *Environ. Sci. Pollut. Res.* **2021**, *28* (32), 44140–44151.

(9) Abdul Hamid, S. B.; Chowdhury, Z. Z.; Zain, S. M. Base Catalytic Approach: A Promising Technique for the Activation of Biochar for Equilibrium Sorption Studies of Copper, Cu(II) Ions in Single Solute System. *Materials (Basel).* **2014**, *7* (4), 2815–2832.

(10) Goswami, R.; Shim, J.; Deka, S.; Kumari, D.; Kataki, R.; Kumar, M. Characterization of Cadmium Removal from Aqueous Solution by Biochar Produced from Ipomoea Fistulosa at Different Pyrolytic Temperatures. *Ecol. Eng.* **2016**, *97*, 444–451.

(11) Ahmed, M. B.; Hasan Johir, M. A.; Zhou, J. L.; Ngo, H. H.; Nghiem, L. D.; Richardson, C.; Moni, M. A.; Bryant, M. R. Activated Carbon Preparation from Biomass Feedstock: Clean Production and Carbon Dioxide Adsorption. *J. Clean. Prod.* **2019**, *225*, 405–413.

(12) Mau, V.; Gross, A. Energy Conversion and Gas Emissions from Production and Combustion of Poultry-Litter-Derived Hydrochar and Biochar. *Appl. Energy* **2018**, *213* (August 2017), 510–519.

(13) Zhang, X.; Gao, B.; Fang, J.; Zou, W.; Dong, L.; Cao, C.; Zhang, J.; Li, Y.; Wang, H. Chemically Activated Hydrochar as an Effective Adsorbent for Volatile Organic Compounds (VOCs). *Chemosphere* **2019**, *218,* 680–686.

(14) Islam, M. A.; Ahmed, M. J.; Khanday, W. A.; Asif, M.; Hameed, B. H. Mesoporous Activated Coconut Shell-Derived Hydrochar Prepared via Hydrothermal Carbonization-NaOH Activation for Methylene Blue Adsorption. *J. Environ. Manage.* **2017**, *203*, 237–244.

(15) Cha, J. S.; Choi, J. C.; Ko, J. H.; Park, Y. K.; Park, S. H.; Jeong, K. E.; Kim, S. S.; Jeon, J. K. The Low-Temperature SCR of NO over Rice Straw and Sewage Sludge Derived Char. *Chem. Eng. J.* **2010**, *156* (2), 321–327.
